# Competitive Reporter Monitored Amplification (CMA) - Quantification of Molecular Targets by Real Time Monitoring of Competitive Reporter Hybridization

**DOI:** 10.1371/journal.pone.0035438

**Published:** 2012-04-23

**Authors:** Thomas Ullrich, Eugen Ermantraut, Torsten Schulz, Katrin Steinmetzer

**Affiliations:** Alere Technologies GmbH, Jena, Germany; Chang Gung University, Taiwan

## Abstract

**Background:**

State of the art molecular diagnostic tests are based on the sensitive detection and quantification of nucleic acids. However, currently established diagnostic tests are characterized by elaborate and expensive technical solutions hindering the development of simple, affordable and compact point-of-care molecular tests.

**Methodology and Principal Findings:**

The described competitive reporter monitored amplification allows the simultaneous amplification and quantification of multiple nucleic acid targets by polymerase chain reaction. Target quantification is accomplished by real-time detection of amplified nucleic acids utilizing a capture probe array and specific reporter probes. The reporter probes are fluorescently labeled oligonucleotides that are complementary to the respective capture probes on the array and to the respective sites of the target nucleic acids in solution. Capture probes and amplified target compete for reporter probes. Increasing amplicon concentration leads to decreased fluorescence signal at the respective capture probe position on the array which is measured after each cycle of amplification. In order to observe reporter probe hybridization in real-time without any additional washing steps, we have developed a mechanical fluorescence background displacement technique.

**Conclusions and Significance:**

The system presented in this paper enables simultaneous detection and quantification of multiple targets. Moreover, the presented fluorescence background displacement technique provides a generic solution for real time monitoring of binding events of fluorescently labelled ligands to surface immobilized probes. With the model assay for the detection of human immunodeficiency virus type 1 and 2 (HIV 1/2), we have been able to observe the amplification kinetics of five targets simultaneously and accommodate two additional hybridization controls with a simple instrument set-up. The ability to accommodate multiple controls and targets into a single assay and to perform the assay on simple and robust instrumentation is a prerequisite for the development of novel molecular point of care tests.

## Introduction

Simultaneous sensitive detection and quantification of multiple molecular targets are key prerequisites for the development of state of the art molecular diagnostic tests [1]–[5]. High sensitivity is required for most diagnostic applications, particularly in the monitoring of blood borne pathogens like human immunodeficiency virus (HIV) or hepatitis-C virus (HCV) [6], [7]. Additionally, it is highly desirable to reduce hands-on-time to a minimum and eliminate the potential for amplicon contamination a significant and widely understated problem [8], [9]. Therefore, the four key requirements for an ideal assay format are high sensitivity, ability to multiplex, quantifiable results and reduced steps requiring an operator.

Molecular target amplification techniques such as polymerase chain reaction (PCR), transcription-mediated amplification (TMA), nucleic acid sequence based amplification (NASBA) and loop-mediated isothermal amplification (LAMP) can provide sensitivity down to the single copy level [10]–[14]. Real time PCR has been established as the gold standard for quantification of target present in a sample [15]–[17]. One particular advantage of real time PCR assays is that they can simultaneously amplify and detect targets. However, the number of targets that can be robustly detected in a single reaction is limited and, therefore, probe arrays have become the preferred method for the detection of multiple targets in a single test [18], [19]. One inherent limitation of assays based on probe array readout is that their workflows require numerous steps to be performed. For target quantification a linear or competitive amplification format is usually used, which substantially limits the sensitivity of the assays. Moreover, amplification, probe-array hybridization, and detection are usually separate steps that require specific reagents and conditions. Reported attempts to combine amplification and quantitative readout on a probe array have been limited by the use of common TaqMan reagents and the inherently limited chemical compatibility of conditions required for solid phase hybridization and bulk volume target amplification [20]. Despite the fact that many devices and assay formats have been developed to integrate the required steps into a single disposable device [21]–[24], none of them have become commercially viable due to the inherent complexity of the assay formats used.

Here we present data on detection and quantification of HIV-1 and HIV-2 RNA with a novel device that was used to simultaneously amplify multiple targets and specifically detect amplification products in real time by competitor monitored amplification. The reported assay starts with purified nucleic acid and is fully self-contained. After loading sample into the reaction chamber, no additional handling or manipulation is required. We have characterized the performance of the assay with samples containing known amounts of HIV-1 and HIV-2 RNA. The system is compared to real time PCR, the commercial gold standard method for viral load testing.

## Results

### Competitive Reporter Monitored Amplification (CMA) - proof of principle

**Figure 1:**
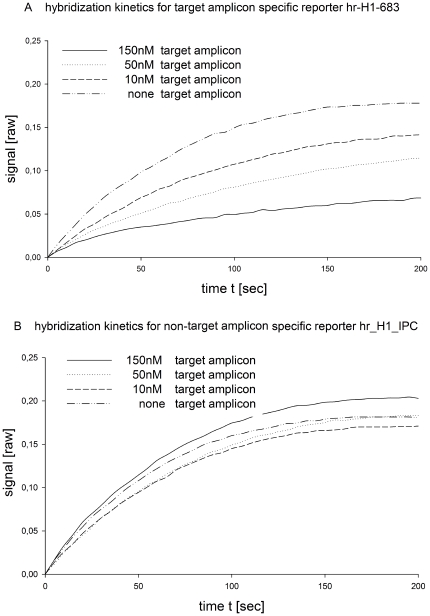
Differences in the hybridization kinetics of a target specific reporter probe and a non-target specific reporter probe. **A.** The diagram shows the kinetics of reporter probe hr-H1-683 in presence of different amounts of hr-H1-683 specific target amplicon. As indicated in the diagram, hybridization speed strongly depends upon the amount of target amplicon present in the reaction. With no target amplicon present in the reaction, reporter probes are available to bind to their complementary capture probes on the array. With increasing amounts of target amplicon, the speed of reporter probe binding to the respective capture probe decreases. Finally, in the presence of approximately the target amplicon concentration expected for a completed PCR- Reaction (150 nM), the reaction speed is at its lowest point. **B.** The data illustrate that the effect is sequence specific. The speed of hybridization of non-target specific reporter probes to their respective capture probes does not depend on the concentration of target amplicons in solution.

The rate of hybridization of a reporter oligonucleotide in solution to a surface immobilized capture oligonucleotide depends on its concentration in solution. The reaction rate can be described as dC[Hybrid]/dt = k*[Reporter]*[Capture Probe], whereby k is the reaction rate constant, [Reporter] the concentration of the reporter probe in solution, [Capture Probe] the concentration of the capture probe and [Hybrid] the concentration of the [Reporter][Capture Probe] hybrid. With increasing amounts of DNA target amplicons in solution, the respective reporter probes bind to these amplicons and the concentration of free reporter probes available for binding to the respective capture probe decreases, leading to a lower hybridization rate for the respective reporter/capture pair. In order to characterize the competitive mechanism of the interactions of the reporter with the reporter specific immobilized capture probes and the reporter specific target amplicons, we used fluorescently labeled reporter probes and monitored the fluorescence intensity of different capture probes on an array. We first determined the hybridization kinetics of the reporter probes to the respective capture probes in the presence of increasing amounts of competitive target DNA while keeping all other reaction parameters constant. To show that the effect on reporter hr-H1-683 is sequence specific, we used a second reporter, hr-H1-IPC, that has a different, non target complementary sequence. Results are shown in **Figure 1**.

**Figure 1A** illustrates that at any given time, the intensity of the fluorescent signal at the respective capture probe position on the array will decrease as the amount of target amplicon in solution increases. This is due to the fact that, with increasing concentration of target amplicon, more reporters are being absorbed by them [25]. Measured signal intensities after only 30 seconds of hybridization range from 0.025 relative intensity units (RIU) for 150 nM target amplicon concentration, 0.034 RIU for 50 nM, 0.044 RIU for 10 nM and 0.064 RIU for no target amplicon in solution, respectively.

As shown in **Figure 1B**, the non-target specific reporter hr-H1-IPC shows signal intensities of 0.0795 RIU, 0.065 RIU, 0.065 RIU and 0.075 RIU, respectively. The fluorescence signal of the hr-H1-IPC reporter probe remains stable, irrespective of target amplicon concentration, indicating that the reporter probe is not reacting with the target present in the solution.

### Hybridization pattern characteristics during a monoplex amplification reaction

**Figure 2:**
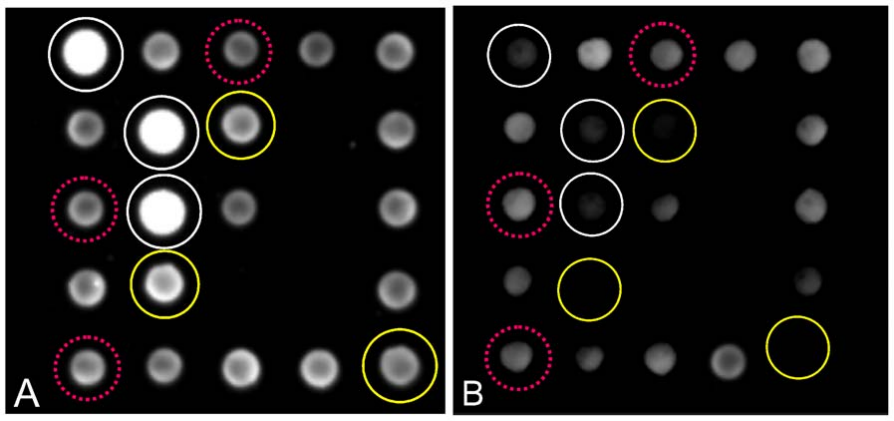
Differences in fluorescence signal intensity on the capture probe array at the first and the last cycle of a monoplex amplification reaction. **A.** An image of a hybridization pattern at the first cycle of the amplification reaction is shown. No target amplicon is present in the solution. The capture probe for the HIV-1 target specific reporter probe hr_H1_683 (solid yellow circle) as well as the non-target specific reporter probes, for example hr_H1_IPC (dotted red circle) show maximal signal intensity under the given reaction conditions. Initially, all reporter probes are free to bind to their respective capture probes on the array and show maximal signal intensity. The capture probe for hr_NHC shows no hybridization signal due to absence of a corresponding reporter probe. **B.** After 45 cycles of amplification, the target specific reporter probes hr_H1_683 are absorbed by the target amplicons and accumulate in the solution phase. The signal is “dragged" from the array into the solution, resulting in decreased fluorescence signal intensity on the complementary capture probe. In contrast, fluorescence signals for non-target specific reporter probes remain almost at their initial levels, for example hr_H1_IPC. In contrast to the hybridization probes, the covalently bound index spots (solid white circle) show significant bleaching effects. Bleaching is not observed on capture probes. This difference is due to the replenishing effect of the cyclic hybridization of reporter probes to the capture probes.

We assessed the achievable shift of hybridization signal intensity detected on the respective capture probe between the first and the last cycles of a monoplex amplification reaction. A HIV-1 PCR amplicon, prepared and quantified as described in the methods section, was used as amplification template with an initial amount of 15,000 copies per reaction. The experiment was carried out by using the cartridge, breadboard setup, process conditions and reaction mixture as described in the Methods Section. A fluorescence image from the hybridization pattern was acquired at the end of the annealing step from the first and the last cycle of amplification. **Figure 2A** shows the image of a CMA hybridization pattern during the first cycle of the amplification reaction. Target specific reporter probe hr_H1_683 as well as the non-target specific reporter probes e.g. hr_H1_IPC are free to bind to their complementary capture probe on the array. A bright fluorescence signal is detectable on all capture probe positions except for the negative hybridization control hr_NHC. During PCR, target is amplified and target specific reporter probes hybridize to their respective target amplicons in solution; thus, the signal for the target specific reporter probe hr_H1_683 decreases while signals for the non-target specific reporter probes, for example hr_H1_IPC, remain constant. **Figure 2B** shows an image of a CMA hybridization pattern during the last cycle of the amplification reaction. By the end of the amplification reaction the signal for the target specific reporter probe hr_H1_683 has visibly decreased to almost the signal intensity of the background whereas the signals for the non-target specific reporter probes are still visible.

**Figure 3:**
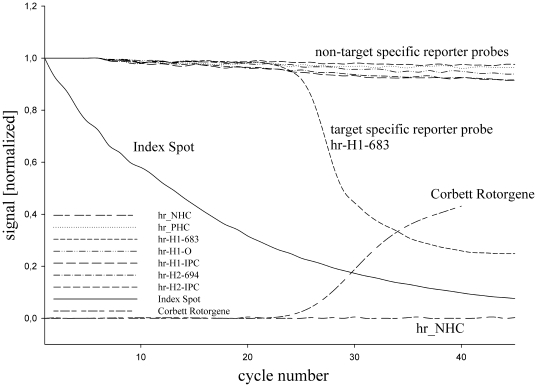
Hybridization signal dynamic on the micro array during a monoplex amplification reaction. The calculated signal intensities for the hybridization pattern of each acquired image were plotted against the cycle number. Images have been acquired after each cycle at the end of the annealing phase. Signal intensity decrease of about 70% can be observed at the HIV-1 target specific reporter probe hr_H1_683. The signal follows a typical sigmoidal dynamic with clearly recognizable phases of a PCR reaction. In contrast, for non- target specific reporter probes e.g. hr_H1_IPC, the decrease of signal intensity is linear and just about 5% from the initial value. Compared to the TaqMan data obtained by a real time PCR using the Corbet Rotorgene instrument, signals in the CMA experiment are discernable from background noise at about the same cycle number as in the reference experiment. Significant differences in bleaching behaviour are observed between fluorophores attached covalently to the surface and those attached to reporter probes.

To assess the signal dynamic on the array throughout an amplification reaction, a fluorescence image from the hybridization pattern was acquired at the end of the annealing phase of each cycle. The signal intensities were calculated for all reporter probes, normalized and plotted against the cycle number. In addition, we amplified the same template amount using the Corbett Rotor Gene 6000 reference system and compared the Taqman based data with the CMA data. The results are shown in **Figure 3**. Signal intensity for target specific reporter probe hr_H1_683 decreased by approximately 70% whereas signal intensities for non-target specific reporter probes only decreased by approximately 5%. The signal for the target specific reporter probe follows a typical sigmoidal curve with the clearly recognizable phases of a PCR reaction. Signals in the CMA experiment are discernable from background noise at about the same cycle number as the reference TaqMan data. In contrast to the target specific probes intensity, non-target specific probe intensity decrease linearly and remain almost constant in absolute terms. The observed linear signal decrease is explained by a gradual loss of a fraction of the immobilized capture probes because of thermal stress and bleaching of the fluorophores due to light exposure.

### Bleaching

Since bleaching is a common and expected occurrence in fluorescence detection techniques, we investigated bleaching characteristics of the reporter probes in our test procedure. In contrast to common detection methodologies based on hybridization, washing procedures and endpoint detection, in our process the hybridization pattern needs to be exposed to light during each amplification cycle. **Figure 2** and **Figure 3** show the difference in bleaching behaviour of fluorescence labels irreversibly attached to a surface and reporter bound fluorescence labels throughout a cyclic amplification reaction. In contrast to the irreversibly attached fluorescence label, hybridized fluorescence labels show far less bleaching. This is due to a turnover of reporter probes during each PCR cycle. As the amplification reaction enters the denaturation phase at 95°C, reporter probes dissociate from the capture probes and from the target amplicon. During the annealing step, some reporter probes hybridize to the immobilized capture probes. However, at each cycle, only a fraction of reporter probes are bound to the capture probes. The vast majority of reporter probes stay in solution, protected from light by the optical aperture. Since it is unlikely that the same reporter probe hybridizes to the immobilized probe again, a replenishing effect on the observed fluorescence intensity is to be expected and is, in fact, observed. In contrast, covalently bound fluorescent labels are exposed to excitation during each cycle and therefore are affected more extensively by bleaching.

### Assessing linearity of the competitive assay

**Figure 4:**
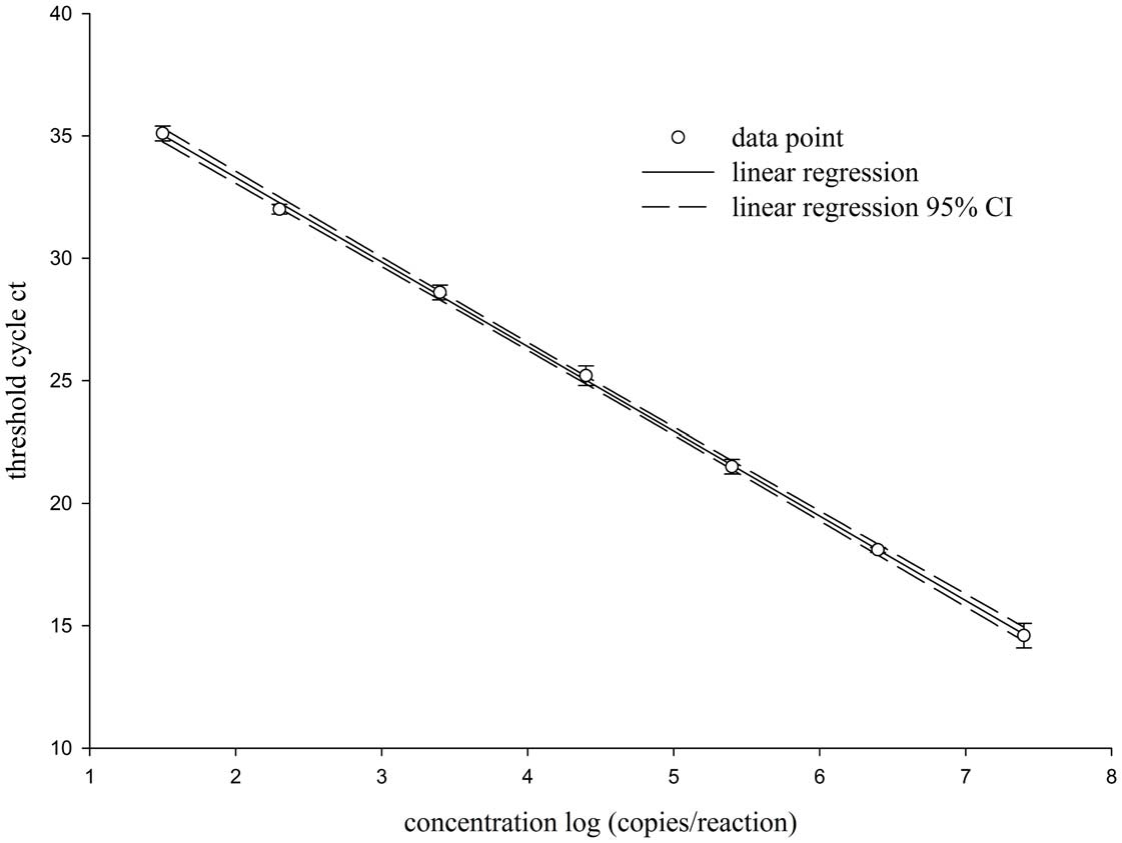
Standard curve obtained by the competitive reporter monitored amplification method. Samples with a known amount of target template have been used to establish a standard curve. The amount of target is given as logarithmic copy numbers as indicated in the graph. The threshold cycle values are plotted against the logarithm of the initial amount of target template copies. The curve function has been determined as C_t_ = −3.55*log (copy numbers)+39.73 (r = 0.999, P<0.001). The amplification efficiency is calculated to be 92%.

The linearity of our competitor monitored amplification was determined by using a serially diluted HIV-1 PCR amplicon as template for the real time PCR reaction. Template concentrations used in the experiments and threshold cycle C_t_- values are summarized in **Table 1**. The C_t_ - value and logarithm of copy numbers were plotted against each other and linear regression analysis, using the method of least squares, was performed. The resulting diagram is shown in **Figure 4**. We found a statistically significant correlation between target concentration and C_t_ - values (r = 0.999, P<0.001). The resulting calibration curve is described by the equation C_t_ = −3.55*log (copies template)+39.78; R^2^ = 0.9962.

**Table 1:** Initial target amount and C_t_ -values for assess the linearity of the CMA.

Target amount (number of copies)	Mean C_t_ - value (n = 4)	Standard deviation (n = 4)
NTC	No signal	---
20	35.1	0.3
140	32.4	0.3
1400	28.6	0.3
14000	25.2	0.4
140000	21.5	0.3
1400000	18.1	0.1
14000000	14.6	0.5

The table show the initial target amount, the mean C_t_ - value and the standard deviation for the linearity experiments. The number of replicates is indicated in the brackets. No C_t_ - value was found for the NTC (no template control).

Amplification efficiency, calculated by using the slope of the calibration curve according to equation **E = 10^(−1/slope)^**, was found to be E = 1.92. Under ideal reaction conditions efficiency should be E = 2. Usually, real-time PCR efficiency varies with high linearity (r>0.989) from E = 1.6 to maximal values up to E = 2.10, for cDNA input ranges from a few pg to 75 ng. Slopes between −3.1 and −3.6 are considered acceptable [26].

### Specificity

We tested the specificity and multiplexing capability of the new format. The specificity was assessed with RNA from different samples containing HIV-1 RNA, HIV-2 RNA or no template control (NTC). Moreover, the ability to discriminate between HIV-1 group M subtype A-J and HIV-1 group O was also assessed. In each reaction the internal process controls (IPC) for HIV-1 as well as for HIV-2 were co-amplified.

In summary 139 samples were analysed. 56 samples contained HIV-1 group M target, 22 samples contained HIV-2 target and 5 samples contained HIV-1 subtype O target. For all samples, RNA concentration was determined by the reference method. 56 experiments were performed without HIV template (NTC). No false positive or false negative results were obtained in any experiment. Within this experiment series, we did not observe non-specific signal decrease on the positive hybridization control (PHC) or unspecific signal formation on the negative hybridization control (NHC). The IPC for the specific target was co-amplified and detected in all reactions with an average C_t_ - value for the HIV-1 IPC of 31.1 and for the HIV- 2 IPC of 31.0 with a standard deviation of 1.2 and 1.4, respectively.

### Method comparison between CMA and standard Taqman real time PCR

**Figure 5:**
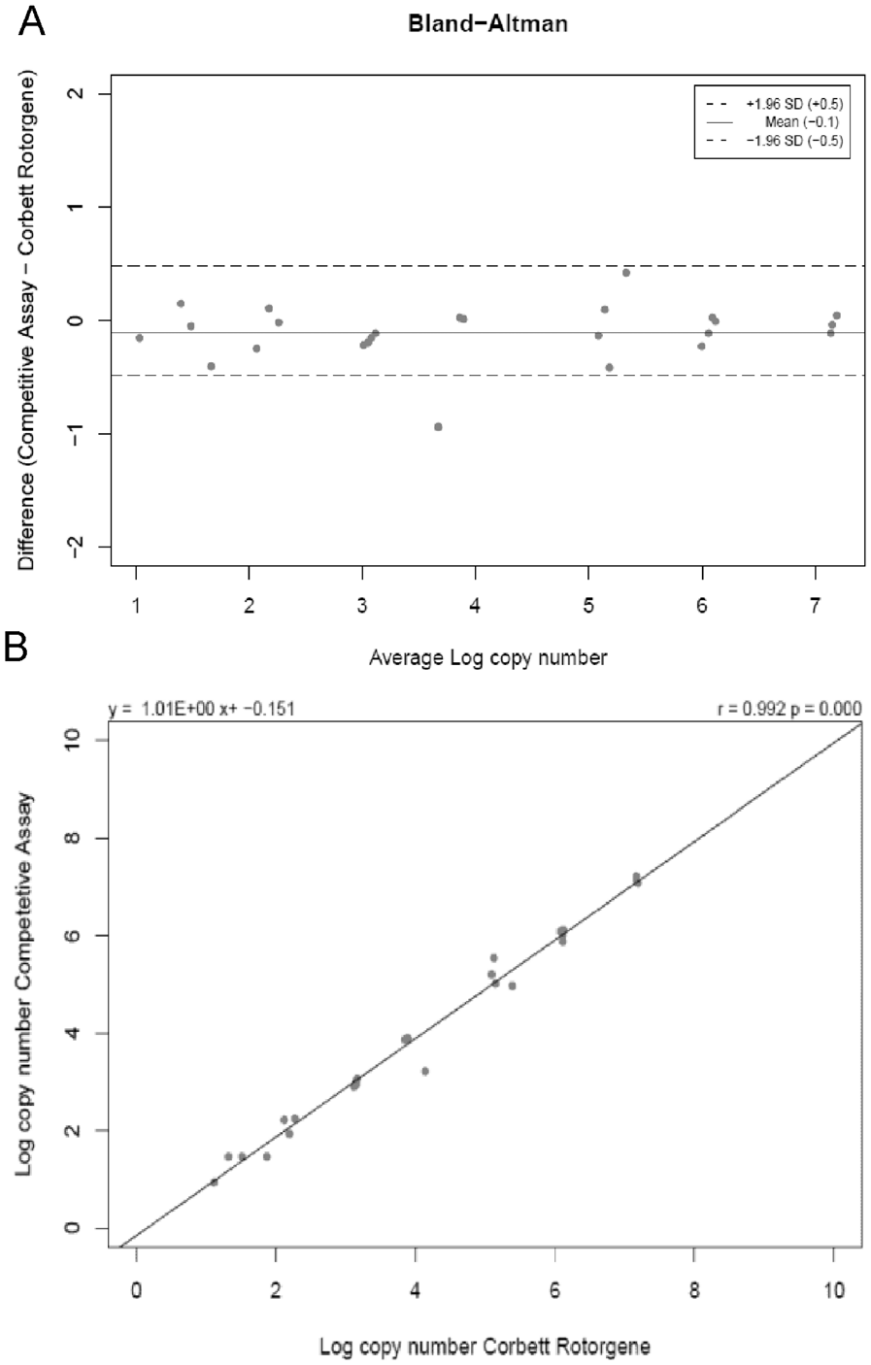
Bland- Altmann analysis of CMA and real time RT-PCR data. **A.** In order to analyse the agreement between competitive reporter monitored amplification CMA and conventional RT-PCR (the reference method), the respective data have been plotted by use of a Bland-Altman Plot. Methods have been found to be in good agreement with limits of agreement at +/−0.5 and a bias of −0.1. **B.** Log copy numbers of the reference method and the competitive assay calculated by their respective calibration curve are compared (n = 25). A strong correlation between the two methods is observed (r = 0.992, P<0.001).

To compare the methods, we performed a set of parallel experiments (n = 25) with the same initial copy number. The data were compared using a Bland-Altman Plot to show the agreement between the two different methods **(** **Figure 5A** **)**. In the plot, we compared logarithmic copy numbers for the reference method with logarithmic copy numbers of the competitive assay, obtained by their respective calibration curves. The median is about zero and indicates no systematic differences between the two methods. The plot shows the 1.96 SD in the range of +/−0.5 log copy number. The mean value is −0.1. A significant correlation was found between the respective, calculated copy numbers from each method (r = 0.992, P<0.001) (**Figure 5B**).

## Discussion

It has been generally recognized that many molecular diagnostics scenarios require detection of multiple targets as well as numerous controls in a single reaction [27], [28]. Moreover, target quantification is also often important, not only to determine the exact number of targets as, for example, markers for disease progression, but also in order to improve assay precision in qualitative assays. Short time to result is also always desirable, especially for point-of-care diagnostics [29]–[31]. We have presented an assay format that meets the outlined requirements and thus allows for the simultaneous, quantitative detection of multiple molecular targets. In our format, exponential target amplification is achieved by conventional polymerase chain reaction. Similar to other homogenous real time detection formats, we use specific reporter probes to interrogate the amplified target region. In our assay, however, all reporter probes are labeled with the same fluorescent label and are complementary to the target region on the amplification target as well as to surface immobilized capture probes. Since the probe array is part of the containment in which the amplification is carried out, the hybridization of reporter probes to capture probes on the array is directly influenced by the amount of amplified target. As more target is amplified more reporter probes hybridize to target with the consequence that less reporter probes hybridize to their respective capture probes on the array.

In order to practically facilitate this format, we have designed and built a device that allows imaging of the fluorescent pattern on the capture probe array without intermediate washing steps and without using sophisticated optical setups. The outlined principles of mechanical displacement of background fluorescence as well as the competitive reporter monitored amplification (CMA) approach are being reported for the first time. With the presented system, the kinetics of the amplification reaction was similar to a conventional real time polymerase chain reaction while utilizing only a single fluorescent label.

With the HIV model assay, we observed the amplification kinetics of five targets simultaneously and accommodated two additional hybridization controls. In total, we used seven individual capture probes, six individual reporter probes and five different primers for target amplification.

Since the amplified target and the immobilized capture probe compete for the reporter probe, the effect on fluorescence signal intensity is immediate and can be observed at a high sampling frequency, reducing time to result. This is a major advantage over hybridizing the amplified target to immobilized probes, as that approach requires a substantial hybridization time. Reporter probes are single-stranded oligonucleotides whereas polymerase chain reaction produces double-stranded amplicons. Since single-stranded nucleic acids hybridize more efficiently with each other, various approaches to generate asymmetric amplification product have been described [32], [33]. Introduction of some form of target asymmetry usually impacts amplification performance and moreover complicates the assay. Also direct hybridization of an amplicon to a surface bound probe requires a certain concentration of amplicon before a fluorescence signal can be measured on the capture probe. Since we have focused on detecting a change in fluorescence on the probe array early in amplification, a competitive approach with short single-stranded oligonucleotides turned out to be advantageous. In other homogenous amplification formats, for example TaqMan, fluorescence at a distinct wavelength is generated in liquid bulk and detected by a fluorescence sensor as the amplicon is formed. Additional targets can only be detected by using other fluorescent dyes with their respective sensors and optical filters. For efficient detection, labeled probes are required to be present in the nanomolar range. It is also challenging to separate fluorescence emission of different labels while keeping the transmission of the filter at a reasonable level to ensure sufficient sensitivity. Notwithstanding, numerous instruments on the market do offer multiple filter sets and thus the potential to detect a variety of labels [34]. The level of multiplexing achieved in commercially available quantitative diagnostic tests is usually duplex, combining amplification of the actual target together with amplification of an internal control. Exceptions are assays where multiple targets are used to detect a single microorganism and crosstalk between the filters is less problematic. The presented approach overcomes these limitations. The experimental setup outlined here allowed us to characterize reporter probe hybridization kinetics in the presence and absence of hybridization targets. From the user's perspective, the test principle is simple and allows for a completely closed workflow, without the need to perform washes or additions of liquids during the test assay as required for other test formats. The temporary and reversible compression of the test chamber during the image acquisition is the only actuator step required in the presented workflow. By mechanically displacing the fluorescent background we are able to acquire a high quality fluorescence image of the hybridization pattern on the array, without using sophisticated optical setups, that is virtually unaffected by the actual background fluorophore concentration.

We applied the new assay format and setup to the development of a multiplex assay for the quantitative detection of HIV-1 and HIV-2 RNA. We assessed the performance of the new format against TaqMan as the standard. The assay showed a linear response in the range from log 1.3 copies to log 7.1 copies (r = 0.999, P<0.001) The calculated amplification efficiency of approximately 92% was in the expected range for a well performing real time PCR amplification reaction. The lowest copy number input of 20 copies of targets per reaction was detected 100% of the time. We made use of the expanded multiplexing capabilities of the new format and integrated amplification and hybridisation controls into the assay. In a total of 139 experiments, we did not detect any non-specific signals on the hybridization process controls. The internal process controls for the specific targets have been co-amplified and were clearly detected in all reactions with average C_t_ - values for the HIV-1 standard of 31.1 and for the HIV- 2 standard of 31.0 with a standard deviation of 1.2 and 1.4, respectively. With an experimental set up including the internal controls and specific targets for HIV-1 Group M, HIV-1 Group O, and HIV-2, we demonstrated 100% specificity of the test for all nucleic acid templates available to us in this study. No detectable cross reaction was observed between the different targets. While in the present model assay we implemented only seven different capture probes, three amplicon specific probes for HIV, two internal process controls and two hybridisation controls, the assay should be easily expandable to include additional targets.

Although we have been working with polymerase chain reaction as the method for amplification, we recognize that other amplification methods may be combined with the detection and quantification scheme that we propose. In addition to the detection of microbiological targets, the format may be expanded to expression level profiling and genotyping. With the new format, it is possible to use multiple capture and reporter probes simultaneously in an assay. The format does not, however, address the inherent limitations that go along with the use of multiple sets of primers and thus the achievable levels of multiplexing and sensitivity depend on the ability to amplify multiple targets without compromising assay sensitivity.

## Materials and Methods

### Description of the assay principle

**Figure 6:**
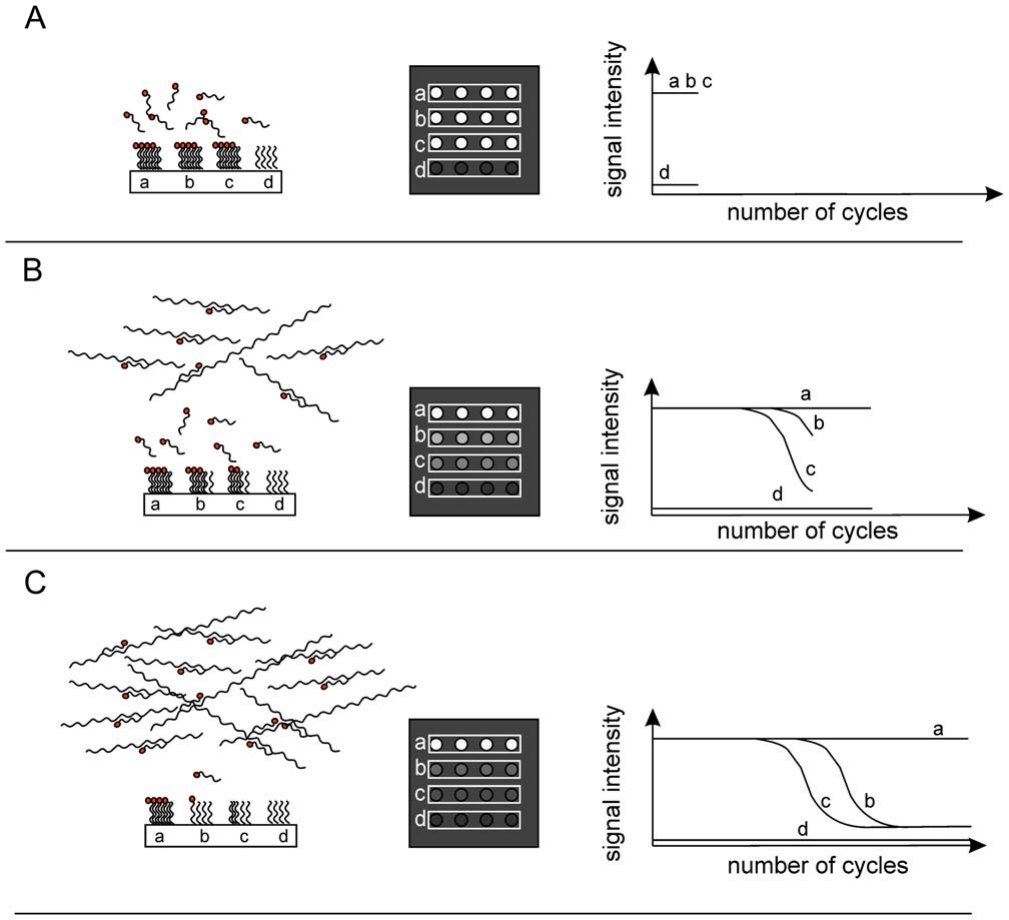
General principle of the competitive reporter monitored amplification (CMA) method. At the onset of the amplification reaction, only a few targets are present. Reporter probes are free to bind to the complementary capture probe on the array **(panel A)**. Hybridization kinetics are driven by the concentration of reporter probes in solution, the amount of the respective surface immobilized capture probes and reaction conditions like temperature and salt concentration. As the amplification reaction proceeds **(panel B)**, more and more target amplicons with a reporter probe specific binding site are synthesized. Along with the accumulation of the target amplicons, the hybridization kinetic becomes dominated by the concentration of target amplicons. As more target amplicons are synthesized, more reporter probes bind to target amplicons. Therefore, the amount of reporter probes hybridized to the complementary capture probes decreases proportionally to the formation of new target amplicons. A decrease in fluorescence signal intensity is observed on the respective position on the capture probe array, until the amplification reaction reaches a plateau **(panel C)**. The change in signal intensity is determined by analysis of the sequential fluorescence patterns on the capture probe array.

The assay principle is based on two main components: an array of immobilized capture oligonucleotide probes and complementary fluorescently labeled reporter oligonucleotide probes in solution. Under suitable conditions, the reporter probes will specifically hybridize to the immobilized capture probes as shown in **Figure 6**. The reporter probes are also complementary to a specific target sequence and its respective amplicons that are generated during PCR and which compete with the immobilized capture probes for the binding of the reporter probes. As shown in **Figure 6A**, at the onset of the amplification reaction, none or only a few targets are present and reporter probes are free to bind to the complementary capture probes on the array. Hybridization kinetics mainly depend on the concentration of reporter probes in solution, the amount of the respective surface immobilized capture probes and reaction conditions like temperature and salt concentration. Throughout the process, hybridization temperature and salt concentration remain constant for each cycle of hybridization.

As the amplification reaction proceeds, more target amplicons with a reporter probe specific binding site are synthesized (**Figure 6B**). As more target amplicons are synthesized, more reporter probes bind to them. The solid support, to which the capture probes are attached, introduces a diffusion barrier which significantly reduces the hybridization rate. Solution phase reactions are kinetically favoured to solid phase reactions [25]. Consequently, the amount of reporter probe hybridized to the complementary capture probe decreases proportionally to the formation of new target amplicons. This decrease can be observed by sequential fluorescence imaging of the capture probe array at each annealing phase of the amplification reaction (**Figure 6C**).

### Description of device and test cartridges

**Figure 7:**
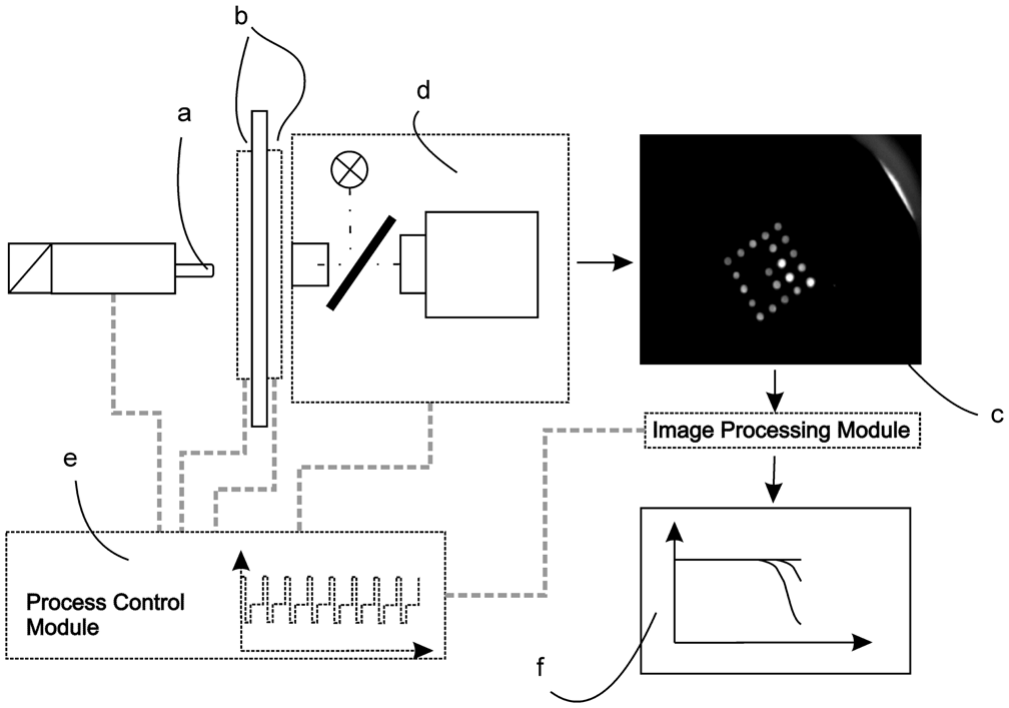
Principal scheme of the test device. The mechanical module contains a plunger (**a**) that is used to reversibly squeeze and release the reaction chamber. The temperature control module comprises two peltier elements with an aperture in the center (**b**). Fluorescence images of the array (**c**) are acquired by the optical detection module (**d**). Synchronized simultaneous performance of all components is controlled by the process control module (**e**), which also controls the thermal regime of the amplification reaction. After image acquisition, the hybridization pattern is analyzed by the image analysis module. Results are visualized as a plot of intensity on each individual probe versus the respective cycle number (**f**).

The device used in this study consists of the following modules: a temperature control module, a mechanical module, an optical detection module, an image analysis module and a process controller module. The principal scheme of the device is shown in **Figure 7**. The temperature control module comprises two Peltier elements for rapidly heating and cooling the reaction chamber. The mechanical module contains a plunger that is used to repeatedly squeeze and release the reaction chamber to press the capture probe array against the polypropylene foil of the reaction chamber for image acquisition. During this process, the bulk liquid is temporarily displaced from the volume over the array. The reaction chamber volume is approximately 100 µl.

The optical detection module integrates a custom made camera equipped with the ICX424 1/3″ CCD sensor (Sony Corporation, Tokyo, Japan), an Aculed VHL Red, ACL01-SC-RRRR-007-C01-L-R000 (Perkin Elmer, Waltham, USA) as the light source, the BrightLine HC 628/40 filter for excitation, BrightLine HC 692/40 filter for emission and the dichroic filter BrightLine BS 660 (all from Semrock, Rochester, USA) into a epi-fluorescence setup. A process control module was designed and software was programmed to operate the system.

**Figure 8:**
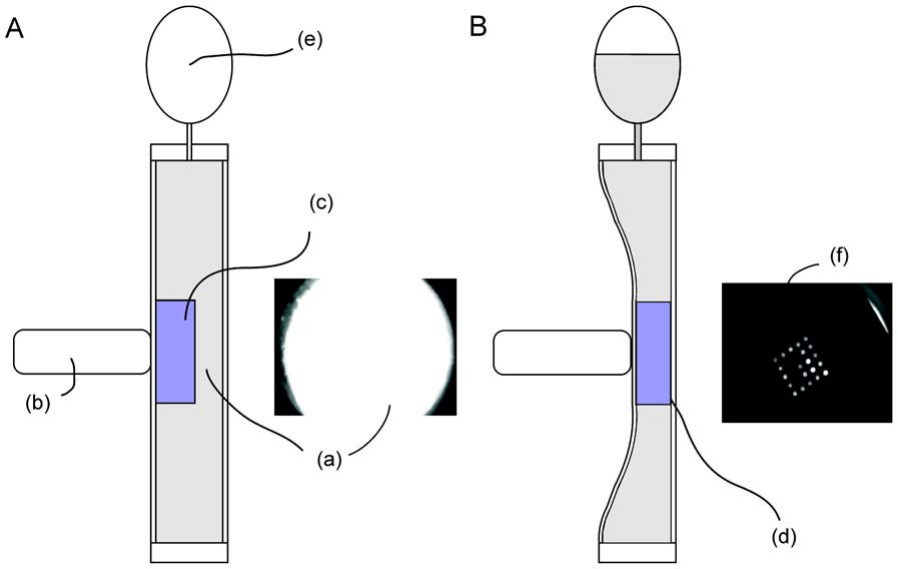
Schematic of the reaction chamber. The uncompressed reaction chamber is filled with the reaction mix containing a high concentration of fluorescently labeled reporter probes. Therefore, imaging of the hybridization pattern on the capture probe array against the background is not feasible (**a**). For image acquisition, pressure by means of a plunger (**b**) is applied and the array (**c**) is pressed tightly against the top side of the reaction chamber, thus displacing the fluorescent liquid (**d**). Displaced liquid is pressed into an integrated pneumatic spring (**e**). After image acquisition (**f**) the chamber is allowed to relax and the liquid mixture returns into the created volume.

In order to detect the fluorescence pattern on the capture probe array and to follow the amplification process in real time, it is necessary to sequentially acquire images after each amplification cycle. To ensure ideal reaction conditions, the capture probe array is completely immersed in the reaction mixture. However, because of the background fluorescence of the reaction mixture caused by the fluorescently labeled reporter probes, in this configuration it is not possible to image the hybridization pattern on the capture probe array. Visualization of the hybridization pattern is facilitated by a mechanical liquid displacement process. For image acquisition, pressure is applied to the reaction chamber and the capture probe array is pressed tightly against one side of the reaction chamber, thus displacing the fluorescent liquid (**Figure 8B**). After fluorescence imaging of the capture probe array, the chamber is allowed to relax and the liquid mixture returns into the created volume.

The cartridge consists of an injection moulded body, forming a reaction chamber with a total reaction volume of 100 µl and a capture probe array. **Figure 8A** illustrates the principal components of the cartridge. The reaction chamber frame has a thickness of 1 mm and is sealed on both sides with a polypropylene foil to define the reaction volume. The capture probe array is embedded into a holder structure and is attached to the polypropylene foil. To facilitate fast heating and cooling cycles, the chamber is brought into tight contact with two Peltier elements which compress the reaction chamber. Thus, optimal thermal contact between the foil and the heater plates is achieved. The reaction mixture enters through an inlet on the bottom of the construction, which is sealed by use of an integrated valve. An outlet at the top of the chamber leads into a pneumatic spring. Once the reaction chamber is filled with the reaction mixture, the air present in the chamber is pressed into the pneumatic spring. Due to the compressibility of the air in the pneumatic spring, it is possible to reversibly compress and decompress the liquid filled reaction chamber. The displaced liquid volume is temporarily pressed into the pneumatic spring.

### Capture probe array

**Figure 9:**
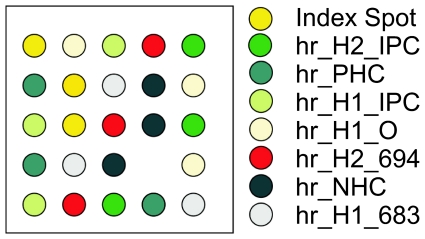
Capture probe array layout. The identifier and the location for the binding side of each reporter probe is indicated. Besides the specific probes for HIV-1 (hr_H1_683), HIV1_O (hr_H1_O) and HIV-2 (hr_H2_694) amplicons, the array contains a positive hybridization control (hr_PHC) and a negative hybridization control (hr_NHC) to assure correct hybridization performance. Moreover, internal process controls for HIV- 1 (hr_H1_IPC) and HIV-2 (hr_H2_IPC) are implemented to confirm amplification performance.

The capture probe arrays used in the experiments were made at the ArrayTube/ArrayStrip facility at Alere Technologies GmbH (Jena, Germany). 3′-Amino end labelled capture probes were obtained from Metabion (Martinsried, Germany). The capture probes were attached to the solid phase according to the layout shown in **Figure 9**. The respective probe sequences are listed in **Table 2**.

**Table 2:** Oligonucleotide sequences of the primers, reporter probes and immobilized capture probes.

Oligonucleotide [Name]	Oligonucleotide [Sequence 5′ to 3′]
pr-H1-634	GCAGTGGCGCCCGAACAGG
pr-H1-809	ACTGACGCTCTCGCACCCATCT
pr-H1-807	TGACGCTCTCGCACCCATCTCTC
pr-H2-640	GCAGGTAGAGCCTGGGTGTTC
pr-H2-760	CTTGCTTCTAAYTGGCAGCTTTATT
hr-H1-683	Cy5-CTCTCGACGCAGGACTCGGCT-Cy5
hr-H1-O	Cy5-CTCCGACGCAACGGGCTCG-Cy5
hr-H1-IPC	Cy5-AGCAGTGGTCAGGCACGAGGAC-Cy5
hr-H2-694	Cy5-TGGGCAGAYGGCTCCACGC-Cy5
hr-H2-IPC	Cy5-CGCACCTCGGCAGACGGGT-Cy5
hr-PHC	Cy5-TCCCAGCGTCCCGTAGTAGTCGG-Cy5
sp-H1-683	AGCCGAGTCCTGCGTCGAGAG-NH_2_
sp-H1-O	CGAGCCCGTTGCGTCGGAG-NH_2_
sp-H1-IPC	GTCCTCGTGCCTGACCACTGCT-NH_2_
sp-H2-694	GCGTGGAGCCRTCTGCCCA-NH_2_
sp-H2-IPC	ACCCGTCTGCCGAGGTGCG-NH_2_
sp-PHC	TCCCAGCGTCCCGTAGTAGTCGG-NH_2_
sp-NHC	TCCGCTACTCGGGATCAGGGAGC-NH_2_

Modifications such as NH2-linker for immobilization and fluorescence labels are shown. H1 and H2 indicate the targets HIV-1 and HIV-2 respectively. Capture probes and reporter probes used to detect the internal process controls for HIV-1 and HIV-2 are labelled as IPC. The sequences for the positive and negative hybridization controls (PHC and NHC) are also provided.

### Reverse Transcription – Polymerase Chain Reaction (RT- PCR)

For the detection of HIV-1, we developed a set of primers and probes covering highly conserved regions in a broad range of sequence variants. The primer design allows discrimination between HIV-1 Group M and Group O. For the detection of HIV-2, primer and probe sequences were taken from Ferns et al. [35] . Primer and reporter sequences used in this study are shown in **Table 2** **.**

The RT- PCR volume in the Cartridge was 100 µl. The reference method was the Corbett Rotorgene System (Qiagen, Hilden, Germany) using the RNA UltraSense One-Step Quantitative RT-PCR Kit (Invitrogen, Carlsbad, CA, USA) and a volume of 50 µl. The final concentration of each primer was 500 nM (Metabion, Martinsried, Germany) and all fluorescently labelled reporter probes were used at a concentration of 20 nM. RT-PCR conditions were: reverse transcription at 50°C for 900 seconds, initial denaturation at 95°C for 120°C seconds, followed by 45 cycles of denaturation at 95°C for 2 seconds, annealing/data acquisition at 60°C for 30 seconds and elongation at 72°C for 30 seconds. The temperature ramp rates were approximately 20 K/sec and 7 K/sec for denaturation and reassociation, respectively.

### Samples

For investigation of hybridization kinetics and linearity studies, PCR product was prepared according to the protocol above with the exception that reporter probes were omitted from the reaction. After PCR, amplicons were purified using a QIAquick PCR Purification Kit (QIAGEN GmbH, Hilden, Germany). Concentrations of purified amplicons were measured by using a Nanodrop UV- VIS ND- 1000 photospectrophotometer (NanoDrop, Wilmington, USA).

HIV-1 RNA was isolated from the Optiquant® HIV-1 RNA quantification panel, obtained from Acrometrix (Acrometrix, Benicia, USA), which is quantified using the World Health Organisation (WHO) HIV-1 International Standard. To verify the discrimination between HIV-1 Group M and HIV-1 Group O, a HIV-1 subtype panel was obtained from the National Reference Centre for Retrovirus (University of Erlangen, Erlangen, Germany). HIV2- RNA was isolated from HIV-2 subtype A strain ROD from cell free culture supernatant obtained from the National Institute for biological Standards & Control (NIBSC), Herts EN6 3QG, UK.

Generally, the RNA was isolated by use of the High Pure RNA Isolation Kit obtained from Roche Diagnostics (Roche Diagnostics Deutschland GmbH, Mannheim, Germany) and amplified using a Corbett Rotor Gene 6000 (Qiagen, Hilden, Germany)

### Hybridization kinetics

All measurements were performed using the cartridge and the device described above. PCR mixes were prepared according to the protocol in the RT- PCR reaction section, with the exception that reporter probes were omitted from the reaction. Processed PCR mixes have been run for 40 cycles whereas non-processed PCR mixes have not been run on a cycler. We prepared a dilution series of a processed PCR reaction mixture in non-processed PCR reaction mixtures with concentrations of 150 nM, 50 nM, 10 nM target amplicon, respectively. The concentration of target amplicon in the processed PCR mix was determined by using a Nanodrop UV- VIS ND- 1000 photospectrophotometer (NanoDrop, Wilmington, USA). In addition we prepared a negative control containing no target amplicon. All dilutions where made to a final volume of 100 µl and included a target specific reporter probe hr-H1-683 and a non-target specific reporter probe hr-H1-IPC at a concentration of 10 nM respectively. Hybridization kinetics were monitored following a protocol consisting of a denaturation step at 95°C for 120 seconds followed by a reassociation step at 65°C for 200 seconds. Fluorescence images were acquired every 5 seconds. The temperature ramp rates were approximately 20 K/sec and 7 K/sec for denaturation and reassociation, respectively.

### Process Controls

#### Positive Hybridization Control (PHC)

For the detection of a potential non-specific decrease of hybridization signal on the capture probe array, we designed a positive hybridization control (PHC), which consists of an immobilized capture probe and its complementary fluorescently labelled reporter probe. Both probes have been selected to have no sequence homology to HIV targets or the internal process controls (IPC). Since no target amplicon is synthesized in solution, the signal on the capture array is expected to remain constant throughout the process. For a valid test, the signal on the PHC needs to remain constant with no reported C_t_ -value, otherwise the test is considered as not valid. Respective capture probe and reporter probe sequences are listed in **Table 2**.

#### Negative Hybridization Control (NHC)

To detect potential non-specific binding of reporter probes to immobilized capture probes, we designed and implemented a negative hybridization control (NHC). For this purpose, we used a capture probe without sequence homology to the HIV target or the internal process controls (IPC) and whose complementary reporter probe was not present in the reaction solution. In the absence of fluorescently labelled complementary reporter probe, no hybridization signal is expected to be detected on this capture probe unless non-specific binding of reporter molecules to the NHC probe occurs. Acceptable NHC signal is defined as a signal intensity which does not exceed a value of 1/3 of the normalized signal for probes with reporters present in the reaction volume. The respective probe sequence is shown in **Table 2**.

#### Internal Process Controls (IPC)

A variety of strategies have been developed to ensure reliable assay performance [36]–[38] and to compensate for adverse effects like inhibition. In our assay, artificial IPC RNA sequences were designed from partial sequences of HIV-1 and HIV-2 in order to ensure proper target amplification and assess the efficiency of RNA amplification. In addition, the incorporation of internal process controls provides a quantification standard. This is particularly useful in applications such as monitoring HIV RNA levels in response to antiretroviral therapy. For both IPC's, transformed *Escherichia coli* DH5a containing the IPC gene (for HIV- 1 and HIV- 2 respectively) cloned into the pCR 2.1 TOPO® vector (Invitrogen, Carlsbad, U.S.A.) were purchased from Eurofins MWG Operon (Ebersberg, Germany). For *in vitro* transcription, plasmid DNA was linearized with HindIII Restriction Endonuclease (Invitrogen, Carlsbad, U.S.A.). *In vitro* transcription and packaging of the transcript (size 500 nucleotides) was done by Asuragen Inc. Armored® RNA technique (Asuragen, Inc. 2150 Woodward St., Suite 100 Austin, TX 78744 USA). The main advantage of Armored RNA® controls is that the packaged RNA shows generally improved stability and is ribonuclease resistant in plasma and other matrices [39].

Primer binding site sequences of the IPC are identical to the target primer binding sites allowing for similar amplification conditions for viral RNA and IPC, whereas the reporter probe binding sites are specific for the IPC to allow discrimination between IPC and the actual target. To avoid competition between target amplicon and IPC, amplicon primers are provided in excess. The respective primer and reporter probe sequences are shown in **Table 2**.

### Reference method and calibration curve

In order to determine the linear range of the CMA format, PCR product was prepared according to the protocol above, with the exception that reporter probes were omitted from the reaction. After PCR, the target amplicons were purified using a QIAquick PCR Purification Kit (QIAGEN GmbH, Hilden, Germany). Concentration of purified target amplicons was determined by using a Nanodrop UV- VIS ND- 1000 spectrophotometer (NanoDrop, Wilmington, USA). Purified target amplicon was then used in serial dilutions as the template in a Corbett Rotor Gene 6000 real time PCR System. Reaction parameters were as outlined before. Based on the obtained data, linear regression was used to generate a calibration curve. In order to calculate copy numbers of the target applied to the reaction mix, the following formula was used: (C_t_) = −3.35*(log copies)+38.11; R^2^ = 0.998). For the direct comparison of both methods, amplification reactions with the same initial target input were performed (n = 25). For both methods, copy numbers were calculated from C_t_ - values by using the calibration curve.

### Data processing, normalization & quantification strategies

Real-time PCR time series can be strongly affected by varying noise signal characteristics [40], [41]. Therefore, an algorithm is required that is able to identify and eliminate different noise signals from data obtained by the CMA method. Our signal processing algorithm is based upon mathematical model fitting; three signal models are involved:

1. An overall signal model for the measured time series
2. A PCR signal model that describes the undisturbed amplification curve (sigmoid function)
3. A noise signal model that describes disturbance effects (linear or exponential function)

The overall signal model combines the model assumption about the undisturbed behavior of amplification data (PCR signal model) and a model assumption about the disturbance effect (disturbance signal model). In an initial processing step of the algorithm, all time series are normalized using the plateau level at the onset of the amplification reaction. Furthermore, all time series are smoothed using a first order median filter.

Subsequently, for each time course, it is determined if target amplicons were hybridized and, if so, which characteristics the disturbance signal follows. For that task, four different alternative overall signal models are adapted to each normalized time series: (a) a second order polynomial, (b) a sigmoid function, (c) a combination of linear and sigmoid function, and (d) a combination of exponential and sigmoid function.

Considering the different model complexities, the best fitting signal model is selected. In case a second order polynomial (a) fits adequately, it is assumed that no amplification occurred. For the other three overall signal models (b)–(d) that include a sigmoid function, it is assumed that the target was amplified and target amplicons were synthesized. Depending upon the selected model type (b)–(d), the influence of the disturbance is either: negligible (b), linear (c) or exponential (d). In an iterative procedure, the lag phase of the amplification time series is determined and, based upon the selected disturbance characteristic, an appropriate disturbance model is derived from that lag phase data. This model is then used to correct the respective real-time PCR time series over the whole time course.

Finally, an amplification signal model (sigmoid function) is identified from the corrected real-time amplification data and the cycle threshold value is estimated from that regression curve, based upon the threshold-line method. In this approach, all signal models are adapted using the Levenberg- Marquardt algorithm.

Threshold- Line and determination of the C_t_ - values for all experiments were set at a value of 0.97 from the maximally normalized fluorescence signal of 1.
